# Emergence of human caliciviruses among diarrhea cases in southwest China

**DOI:** 10.1186/s12879-016-1831-5

**Published:** 2016-09-23

**Authors:** Shun-Xian Zhang, Li Li, Jian-Wen Yin, Miao Jin, Xiang-Yu Kong, Li-Li Pang, Yong-Kang Zhou, Li-Guang Tian, Jia-Xu Chen, Xiao-Nong Zhou

**Affiliations:** 1 National Institute of Parasitic Diseases, Chinese Center for Disease Control and Prevention, Shanghai, 200025 People’s Republic of China; 2 Key Laboratory of Parasite and Vector Biology, Ministry of Health of China, WHO Collaborating Center for Tropical Diseases, National Center for International Research on Tropical Diseases, Shanghai, 200025 People’s Republic of China; 3 The First People’s Hospital of Yunnan Province, Kunming, 650000 People’s Republic of China; 4 Yunnan Provincial Center for Disease Control and Prevention, Kunming, 650000 People’s Republic of China; 5 National Institute for Viral Disease Control and Prevention, Chinese Center for Disease Control and Prevention, Beijing, 102206 People’s Republic of China; 6 The First Clinical Medical College of Lanzhou University, Lanzhou, 730000 People’s Republic of China

**Keywords:** Acute diarrhea, Epidemiology, Sporadic cases, Calicivirus, Norovirus, Novel genotype

## Abstract

**Background:**

Acute diarrhea is one of the most serious problems in global public health that causes considerable morbidity and mortality worldwide. Human caliciviruses (HuCV) including norovirus (NoV, genogroup GI and GII) and sapovirus (SaV), is a leading cause of acute sporadic diarrhea in individuals across all age groups. However, few studies had been conducted clarifying the characteristics of HuCV in diarrhea cases across all age groups in China. Our study was aimed at assessing the HuCV-related diarrhea burden and NoV genotypes distribution in southwest China.

**Methods:**

The study was conducted in four hospitals in Kunming city, Yunnan province, from June 2014 to July 2015. Stool specimens were collected from 1,121 diarrhea cases and 319 healthy controls in outpatient departments. Reverse transcription polymerase chain reaction (RT-PCR) was used to detect NoV (GI, GII) and SaV. Sequencing was applied to confirm the three viral infections and phylogenetic analysis was performed to determine their genotypes. A structured questionnaire was used to record the demographic information and clinical symptoms of subjects.

**Results:**

HuCV was detected at an 11.0 % infection rate in 1,121 diarrhea cases and at 3.4 % rate in 319 non-diarrhea subjects (*p* < 0.0001, OR = 3.5, 95 % CI 1.8–6.5). The prevalence of the NoV genogroup GII and genotype GII.4 in diarrhea cases was significantly higher than that found in healthy controls (*p* < 0.0001, *p* = 0.018, respectively). NoV GII (*n* = 118, 10.5 %) was the most common HuCV subtype in diarrhea cases, followed by SaV (*n* = 3, 0.3 %) and NoV GI (*n* = 2, 0.2 %). Of 118 NoV GII strains isolated from diarrhea patients. GII.4 (*n* = 55, 46.6 %) was the predominant strain, followed by GII.3 (*n* = 28, 23.7 %), GII.12 (*n* = 25, 21.2 %), GII.17 (*n* = 8, 6.8 %), and GII.5 (*n* = 2, 1.7 %). Of the 55 GII.4 strains, the GII.4 Sydney 2012 variant had absolutely predominant prevalence (*n* = 52, 94.5 %), followed by the NoV GII.4-2006b variant (*n* = 3, 5.5 %). The GII.4 Orleans 2009 variant was not found in diarrhea cases of the study.

**Conclusions:**

NoV GII was the major genogroup and GII.4 was the most predominant strain detected in diarrhea patients. The GII.17 is an emergent variant in sporadic diarrhea and might become the predominant strain in diarrhea cases in the near future. Rapid, accurate detection kits need to be developed to help us find and treat NoV-associated diarrhea in clinical settings in a timely manner.

## Background

Acute gastroenteritis is still one of the most concerning public health problems that causes considerable morbidity and mortality worldwide [1, 2]. Human caliciviruses (HuCV) including norovirus (NoV) and sapovirus (SaV) are single-stranded RNA viruses [3]. NoV is classified into six genogroups (GI–GVI), which are subdivided into at least 38 genotypes based on their cap and pol genes [4]. NoV is the leading cause of sporadic cases and outbreaks of acute diarrhea worldwide [5, 6]. NoV-related viral gastroenteritis, known as the stomach flu, is one kind of mild diarrhea, and is characterized by sudden intense vomiting (more common in children), watery stool, or liquid stool without blood (more common in adults) [7]. The NoV infection is self-limiting and the symptoms may disappear after less than three days, but prolonged diarrhea symptoms (e.g. dehydration and death) and viral shedding might occur in immune-deficient individuals and the elderly [5, 8].

NoV is one of the important enteropathogens associated with acute diarrhea, but it has been underestimated in terms of its burden for many years, for a lack of adequate diagnostic tools until 1993 [8]. Although NoV had not been cultivated with cells until now, modern biotechnology, including gene sequencing, reverse transcription polymerase chain reaction (RT-PCR), and real time RT-PCR have been applied to detect NoV in fecal specimens. These can help us to accurately understand and estimate the impact of NoV [8, 9].

The prevalence of NoV is about 18.0 % in acute sporadic diarrhea cases worldwide [10]. The detection rates of NoV were fluctuant in different medical facilities, and the prevalence of NoV in community cases (24.0 %) is higher than that of outpatient cases (20.0 %) and inpatient cases (17.0 %), respectively [10]. Simultaneously, NoV had a high prevalence in the Eastern Mediterranean, and Middle and South American regions [11]. NoV-associated diarrhea prevalence is up to 40–640 cases per 10,000 persons in outpatient treatment and 14 per 10,000 persons in emergency department visits in some developed regions [12]. NoV lead more than 21 million sporadic diarrhea cases, over 70 thousands sporadic hospitalization cases, and nearly 150 deaths of all ages, in the United States annually [13, 14]. In addition, economic loss caused by NoV was also serious; more than $284 million in medical charges are induced by NoV infection in emergency departments annually [12].

NoV is also known as one of the major leading causes of acute nonbacterial outbreaks of diarrhea. NoV-associated diarrhea outbreaks occurred more than 900 times during the period of 1993–2011 [15], and involved many diarrhea cases in a short timeframe, causing social panic and anxiety. Outbreaks of diarrhea episodes caused by NoV often occurred in a variety of settings, such as ships, the army, schools, hospitals, and elderly homes [16–19]. Children, soldiers, and the elderly were susceptible to NoV outbreaks [8, 20]. In developing countries, malnourished children or infants raised without good hygienic conditions had a high risk of suffering morbidity and mortality caused by NoV [21].

NoV has caused increasing concern in recent years, which has been attributed to a large number of reported outbreaks of diarrhea and to food safety issues [13, 15]. Acute gastroenteritis is still one of the major public health problems in China [22], with NoV playing an important role in diarrhea, especially in adults [23]. But, overwhelmingly, diarrhea studies have mostly been focusing on rotavirus in young children. Notably, the epidemic characteristics of NoV in diarrhea cases across all age groups is sparsely characterized, especially in low-income regions such as southwest China. The information on distribution of HuCV subtypes and NoV genotypes is limited.

This study aims to: (i) understand the detection rate of HuCV, (ii) grasp the NoV GII genotype distribution, and (iii) understand the relationship between NoV genotypes and clinical symptoms.

## Methods

### Definition of subjects

An “acute diarrhea case” was defined as any case in which the patient excreted stool more than three times within a 24-hour period and in which the stool form was not normal (e.g. loose, watery, bloody, and/or mucousy stool). A “healthy control” was defined as any case in which the subject was without diarrhea symptom and other gastrointestinal diseases.

### Collection of stool specimens and basic information

Four sentinel hospitals were selected for this study, namely the First People’s Hospital of Yunnan Province, the First Affiliated Hospital of Kunming Medical University, the Kunming Children’s Hospital, and Pushan Community Hospital.

Stool specimens were collected from subjects with acute diarrhea and without diarrhea by trained doctors or nurses. Sterilized sampling cups were used to collect stools, the amount of fecal samples was more than 3 g or 3 mL, each stool sample was temporarily preserved at −20 °C and was transferred to the laboratory of the local Center for Disease Control, and then a part of each stool specimen was taken out and balanced to 10 % suspension with a phosphate buffer solution. RT-PCR was applied to examine SaV, and NoV GI and GI. A structured questionnaire was used to record the demographic information and clinical symptoms of each subject. This study was conducted from July 2014 to June 2015.

### Viral RNA extraction

A viral nucleic acid extraction kit (Geneaid Biotech Ltd, Taiwan, China) was applied to extract the viral nucleic acid from each stool specimen following the manufacturer’s protocol. A 15 % fecal suspension was centrifuged for 5 min at 14,000 g, 150 μL supernatant was used to extract the three viral nucleic acids, and 50 μL extracted RNA was obtained and stored at −70 °C prior to virus detection.

### SaV and NoV (GI, GII) detection

A two-step multiplex RT-PCR was performed to detect SaV, and NoV GI and GII. The primes of p289/p290 target the polymerase region as previously described, this pair of primes produces PCR products of 331 bp for SaV and 319 bp for NoV (GI, GII) [24, 25]. The reverse transcription reaction was performed using the SuperScript II kit (Invitrogen, Carlsbad, CA, USA). The reaction condition of cDNA synthesis was 42 °C for 90 min, and 99 °C for 5 min. Each 40 μL reaction volume consisted of 20 μL SYBR® Premix Ex Taq™ (Takara Bio Inc, Shlga, Japan), 2 μL cDNA template, 12 μL DNase and RNase free water, and 1 μL of 10 μM forward primer and reverse primer, respectively. The thermal profile consisted of 94 °C for 5 min, 40 cycles at 94 °C for 70s, 49 °C for 70s, and 72 °C for 1 min, followed by 72 °C for 10 min. The PCR amplicons were analyzed in a 1 % agarose gel electrophoresis at 120 V for 40 min and observed under UV light after ethidium bromide staining. Positive PCR products were kept at −70 °C, and were then sequenced and phylogenetically analyzed.

### Statistical analysis

Data analysis was performed using the Statistical Product and Service Solutions (SPSS v19.0) software (IBM, USA). The odds ratios (ORs) and 95 % confidence intervals (CIs) of categorical variables were calculated using the chi-square test or the Fisher’s exact test with two-tailed. A value of *p* < 0.05 was considered to be statistically significant.

A molecular evolutionary genetics analysis was employed to perform the phylogenetic analysis (MEGA v5.2). A phylogenetic tree was constructed using the neighbor-joining method with 1,000 bootstrap replicates. Reference strains were downloaded from GenBank.

## Results

### Basic information and clinical symptoms of subjects

In the present study, which was conducted from July 2014 to June 2015, 1,440 subjects including children and adults participated. Among those, 1,121 were acute diarrhea patients and 319 were healthy controls. The male to female sex ratios in diarrhea cases and healthy controls were 1.00 and 1.01, respectively (*χ*^2^ = 0.341, *p* = 0.558). The average ages of patients and healthy controls were 0.6 years and 2.3 years, respectively. The majority of the subjects were from urban areas; 753 cases (67.2 %) and 223 (57.0 %) controls. The average frequency of diarrhea in patients was six episodes (quartile range = 2). Various types of clinical symptoms were observed in the diarrhea cases. Vomiting (*n* = 263, 23.5 %) was the most common clinical symptom, followed by fever (*n* = 136, 12.1 %) and dehydration (*n* = 64, 5.7 %).

### The prevalence of HuCV subtypes and NoV GII genotypes in diarrhea cases and healthy controls

The detection rate of HuCV was 11.0 % (*n* = 123) in diarrhea cases across all age groups. Among these, the prevalence of NoV GII was 10.5 % (*n* = 118) in acute gastroenteritis patients of all ages, followed by SaV (0.3 %, *n* = 3) and NoV GI (0.2 %, *n* = 2). The prevalence of both NoV genogroups (GI and GII) was 10.7 % (*n* = 120) in diarrhea cases across all ages.

HuCV was detected in 123 (11.0 %) diarrhea cases and 11 (3.4 %) healthy controls (*p* < 0.0001, OR = 3.5, 95 % CI 1.8–6.5). No significant differences were observed in NoV GI between diarrhea patients (*n* = 2, 0.2 %) and healthy controls (*n* = 1, 0.3 %, *p* = 0.528). SaV was detected in three (0.3 %) diarrhea cases, but was not found in controls (*p* = 0.472). NoV GII was more prevalent in patients relative to healthy controls across all age groups (10.5 %, 3.1 %, respectively, OR = 3.6, 95 % CI 1.8–7.0). NoV genotype GII.4 was found in 55 (4.9 %) diarrhea cases and six (1.9 %) non-diarrhea cases (*p* = 0.018, OR = 2.7, 95 % CI 1.1–6.3). GII.12 was detected in 25 diarrhea cases and three healthy controls (2.2 %, 0.9 %, respectively, *p* = 0.141, OR = 2.4, 95 % CI 0.7–8.0). The detection rate of GII.5 in diarrhea cases was as high as in healthy controls (0.2 %, 0.3 %, respectively, *p* = 0.403, OR = 0.57, 95 % CI 0.05–6.3). No significant differences in GII.12 and GII.5 were observed between diarrhea patients and healthy patients (see Table 1). GII.17 was only found in diarrhea cases (*n* = 8, 0.7 %).

**Table 1:** HuCV infection detected in the stool samples of patients with diarrhea and healthy controls in Kunming, China (age stratified)

Age group	Subtypes/genotypes	Diarrhea	Control	*p-*value	OR (95 % CI)
		N (%)	N (%)		
All age groups Cases = 1,121 Controls = 319	HuCV	123 (11.0)	11 (3.4)	**<0.0001**	3.5 (1.8–6.5)
	NoV GII	118 (10.5)	10 (3.1)	**<0.0001**	3.6 (1.8–7.0)
	NoV GI	2 (0.2)	1 (0.3)	0.528	
	SaV	3 (0.3)	0 (0.0)	0.472	
	GII.4	55 (4.9)	6 (1.9)	**0.018**	2.7 (1.1–6.3)
	GII.3	28 (2.5)	0 (0.0)	**0.001**	
	GII.12	25 (2.2)	3 (0.9)	0.141	2.4 (0.7–8.0)
	GII.17	8 (0.7)	0 (0.0)	0.134	
	GII.5	2 (0.2)	1 (0.3)	0.403	0.57 (0.05–6.3)
0–5 years Cases = 850 Controls = 170	HuCV	98 (11.5)	8 (4.7)	**0.008**	2.6 (1.3–5.5)
	NoV GII	94 (11.1)	8 (5.5)	**0.012**	2.5 (1.2–5.3)
	NoV GI	1 (0.1)	0 (0.0)	0.833	
	SaV	3 (0.4)	0 (0.0)	0.578	
5–65 years Cases = 244 Controls = 137	HuCV	21 (8.6)	2 (1.5)	**0.005**	6.4 (1.5–27.5)
	NoV GII	20 (8.2)	2 (1.5)	**0.007**	6.0 (1.4–26.2)
	NoV GI	1 (0.4)	0 (0.0)	0.640	
	SaV	0 (0.0)	0 (0.0)		
≥65 years Cases = 27 Controls = 12	HuCV	4 (14.8)	1 (8.3)	0.357	1.9 (0.2–19.2)
	NoV GII	4 (14.8)	0 (0.0)	0.213	
	NoV GI	0 (0.0)	1 (8.3)	0.308	
	SaV	0 (0.0)	0 (0.0)		

Significant difference are marked in bold

In patients aged below five years, HuCV and NoV GII were detected more often in those with diarrhea than in those without (11.5 % vs. 4.7 %, respectively, *p* < 0.05, OR = 2.6, 95 % CI 1.3–5.5; 11.1 % vs. 5.5 %, respectively, *p* < 0.05, OR = 2.5, 95 % CI 1.2–5.3). HuCV was more prevalent in patients than in controls aged above five years (8.6 % vs. 1.5 %, respectively, *p* = 0.005, OR = 6.4, 95 % CI 1.5–27.5). NoV GII was more prevalent in patients with diarrhea relative to healthy controls aged above five years (8.2 % vs. 1.5 %, respectively, *p* = 0.01007, OR = 6.0, 95 % CI 1.4–26.2). NoV GII was detected in 14.8 % of elderly cases, but neither NoV GI nor SaV was detected in controls over 65 years. No significant differences in these three virus pathogens were observed between cases and controls over 65 years of age (see Table 1).

One hundred and twenty-three strains of HuCV were isolated from diarrhea cases of all ages. NoV GII (*n* = 118, 10.5 %) was the leading subtype of HuCV, followed by SaV (*n* = 3, 0.3 %) and NoV GI (*n* = 2, 0.2 %). Of the 118 strains of NoV GII isolated from 1,121 diarrhea cases, the predominant genotype was GII.4 (*n* = 55, 46.6 %), followed by GII.3 (*n* = 28, 23.7 %) and GII.12 (*n* = 25, 21.2 %). The prevalences of GII.17 and GII.5 were less than 1.0 % in diarrhea cases. Among the 10 strains of NoV GII detected in healthy controls, the top two most prevalent genotypes were GII.12 (*n* = 3, 0.9 %) and GII.5 (*n* = 3, 0.3 %). Others genotypes were not detected in asymptomatic individuals (see Table 1).

### The age distribution of HuCV subtypes and NoV GII genotypes in diarrhea cases

In the present study, diarrhea cases were divided into five subunits, where 791 (70.6 %), 59 (5.3 %), 21 (1.9 %), 223 (19.9 %), and 27 (2.4 %) diarrhea patients fell into the age ranges of 0–2 years, 2–5 years, 5–14 years, 14–65 years, and over 65 years, respectively. Although NoV GII was more prevalent in children below two years of age and adults above 65 years of age, no significant difference in presence of NoV GII was found between the age groups (*χ*^2^ = 8.31, *p* = 0.061). GII.12 had an obvious age distribution trend (*χ*^2^ = 15.4, *p* = 0.004), which was only found in patients aged 0–2 years (3.0 %) and 2–5 years (1.7 %). The prevalence of GII.4 had not age distribution trend (*χ*^2^ = 3.83, *p* = 0.426). No significant differences in GII.17, GII.3, and GII.5 were observed in diarrhea cases across all age groups (see Table 2).

**Table 2:** The prevalence of HuCV subtypes and NoV GII genotypes in diarrhea cases, by age group

HuCV subtypes and NoV II genotypes	Total *n* = 1,121 N (%)	<2 years *n* = 791 N (%)	2–5 years *n* = 59 N (%)	5–14 years *n* = 21 N (%)	14–65 years *n* = 223 N (%)	≥65 years *n* = 27 N (%)	*p-*value
HuCV	123 (11.0)	94 (11.9)	4 (6.8)	2 (9.5)	19 (8.5)	4 (14.8)	0.178
NoV GI	2 (0.2)	0 (0.0)	1 (1.7)	0 (0.0)	1 (0.4)	0 (0.0)	0.193
SaV	3 (0.3)	2 (0.3)	1 (1.7)	0 (0.0)	0 (0.0)	0 (0.0)	0.489
NoV GII	118 (10.5)	92 (11.6)	2 (3.4)	2 (9.5)	18 (8.7)	4 (14.8)	0.063
GII.4	55 (4.9)	42 (5.3)	1 (1.7)	2 (9.5)	9 (4.0)	1 (3.7)	0.426
GII.3	28 (2.5)	20 (2.5)	0 (0.0)	0 (0.0)	6 (2.7)	2 (7.4)	0.154
GII.12	25 (2.2)	24 (3.0)	1 (1.7)	0 (0.0)	0 (0.0)	0 (0.0)	**0.004**
GII.17	8 (0.7)	5 (0.6)	0 (0.0)	0 (0.0)	2 (0.9)	1 (3.7)	0.503
GII.5	2 (0.2)	1 (0.1)	0 (0.0)	0 (0.0)	1 (0.5)	0 (0.0)	0.901

Significant differences are marked in bold

### The seasonal prevalence of HuCV subtypes and NoV GII genotypes in diarrhea cases

No notable seasonal trend was observed in NoV GI and SaV, but NoV GII (*n* = 39, 14.6 %) had a peak in prevalence in the fall (*χ*^2^ = 12.2, *p* = 0.007). The prevalences of GII.5 and GII.12 did not show distinct seasonal variation, and the prevalences of GII.3 and GII.17 showed minor seasonal variations across one year, respectively. GII.4 was detected throughout the year and significant differences were observed in different seasons (*χ*^2^ = 13.3, *p* = 0.004), with a seasonal peak of GII.4 (*n* = 23, 8.4 %) in the fall (see Table 3).

**Table 3:** Seasonal distribution of HuCV subtypes and GII genotypes in diarrhea cases

HuCV subtypes and NoV genotypes	Spring (Feb–Apr) *n* = 267 N (%)	Summer (May–Jul) *n* = 200 N (%)	Fall (Aug–Oct) *n* = 260 N (%)	Winter (Nov–Jan) *n* = 394 N (%)	*p*-value
HuCV	22 (8.2)	28 (14.0)	41 (15.8)	32 (8.1)	**0.005**
NoV GI	1 (0.4)	0 (0.0)	1 (0.4)	0 (0.0)	0.388
SaV	1 (0.4)	1 (0.5)	1 (0.4)	0 (0.0)	0.448
NoV GII	20 (7.5)	27 (13.5)	39 (14.6)	32 (8.1)	**0.007**
GII.4	8 (3.0)	12 (6.0)	23 (8.4)	12 (3.0)	**0.004**
GII.3	6 (2.4)	7 (3.5)	4 (1.5)	11 (2.8)	0.557
GII.12	4 (1.5)	5 (2.5)	11 (4.2)	5 (1.3)	0.087
GII.17	1 (0.4)	3 (1.5)	1 (0.4)	3 (0.7)	0.505
GII.5	1 (0.4)	0 (0.0)	0 (0.0)	1 (0.3)	0.554

Significant differences are marked in bold

### The clinical symptoms of diarrhea patients with HuCV subtypes and NoV GII genotypes

The various clinical symptoms associated with HuCV subtypes and NoV genotypes are summarized in Table 4. In acute diarrhea cases; vomiting was observed more frequently in diarrhea cases infected with GII.4 (40.0 %, *p* < 0.05), GII.3 (57.1 %, *p* < 0.05), and GII.12 (48.0 %, *p* < 0.05); and dehydration was only associated with GII.4 (14.5 %, *p* < 0.05) and GII.3 (25 %, *p* < 0.05). Watery stool was more commonly observed in diarrhea patients infected with GII.4, GII.3, and GII.12 (63.6 %, 75.0 %, 72.0 %, respectively, *p* < 0.05); and mucousy stool was found less frequently in GII.3 and GII.12 (25.0 %, 24.0 %, respectively, *p* < 0.05) than in GII.4 (see Table 4).

**Table 4:** Clinical characteristics of acute diarrhea cases infected with NoV GII genotypes

Symptom	GII.4		GII.3		GII.12		GII.17	
	*n* = 55		*n* = 28		*n* = 25		*n* = 8	
	N (%)	*p-*value	N (%)	*p-*value	N (%)	*p-*value	N (%)	*p*-value
Fever (>37.3 °C)	10 (18.2)	0.174	2 (7.1)	0.581	3 (12.0)	1.000	0 (0.0)	0.606
Vomiting	22 (40.0)	**<0.05**	16 (57.1)	**<0.05**	12 (48.0)	**<0.05**	2 (25.0)	**<0.05**
Dehydration	8 (14.5)	**<0.05**	7 (25.0)	**<0.05**	3 (12.0)	0.35	1 (12.5)	0.376
Watery stool	35 (63.6)	**<0.05**	21 (75.0)	**<0.05**	18 (72.0)	**<0.05**	5 (62.5)	0.481
Mucousy stool	17 (30.9)	0.400	7 (25.0)	**<0.05**	6 (24.0)	**<0.05**	3 (37.5)	0.727
Other stool	3 (5.0)	1.000	0 (0.0)	0.372	1 (4.0)	1.000	0 (0.0)	1.000

Significant differences are marked in bold

### Distribution of NoV GII genotypes

In the study, 123 strains of HuCV were isolated from acute gastroenteritis cases, of which two (1.6 %) cases were NoV GI, three (2.4 %) cases were classified as SaV, and 118 (95.9 %) cases were classified as NoV GII. Among the 118 strains of NoV GII, five genotypes were identified as followed: GII.4 (*n* = 55, 46.6 %), GII.3 (*n* = 28, 23.7 %), GII.12 (*n* = 25, 21.2 %), GII.17 (*n* = 8, 6.8 %), and GII.5 (*n* = 2, 1.7 %). Of all the 55 strains of GII.4, the GII.4 Sydney 2012 variant was absolutely predominant (*n* = 52, 94.5 %), followed by the GII.4-2006b variant (*n* = 3, 5.5 %). The GII.4 Orleans 2009 variant was not found in diarrhea cases in this study.

**Fig. 1:**
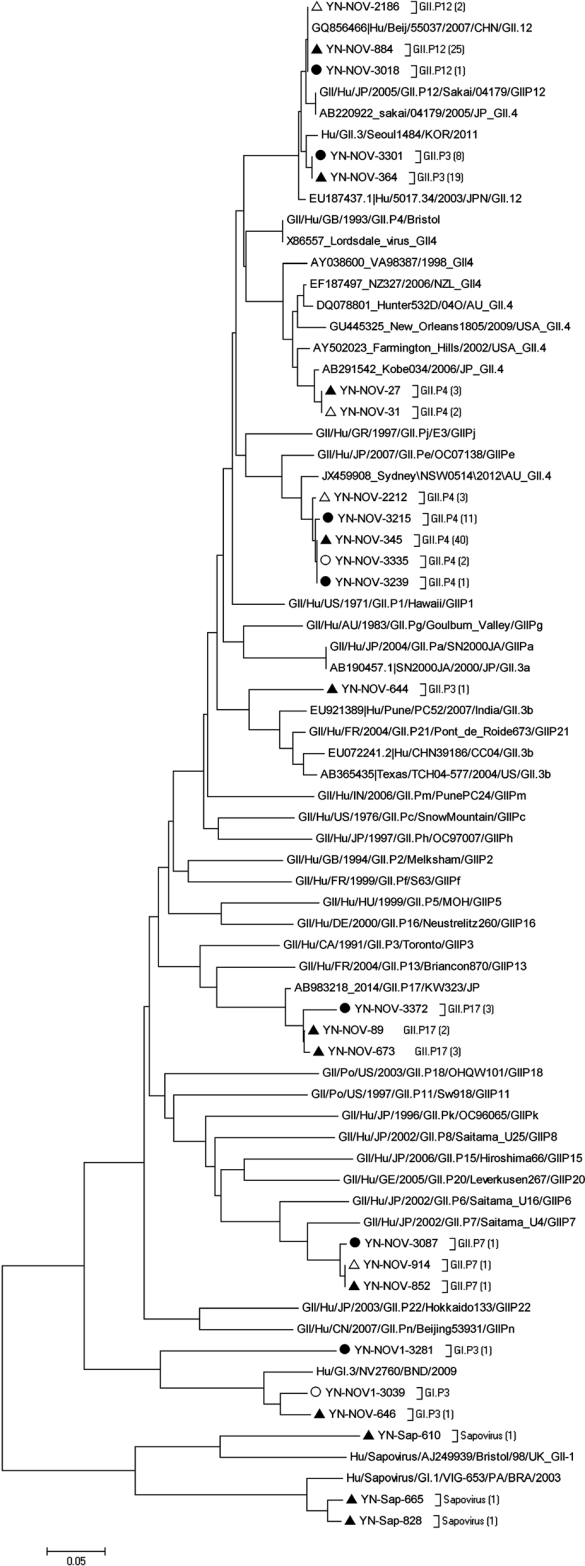
Phylogenetic trees of SaV and NoV (GI, GII) based on dependent RNA polymerase. ●: diarrhea cases above five years of age; ○: controls above five years of age; ▲: diarrhea cases below five years of age; △: controls below five years of age. The count in bracket is the number of same genotype isolated from diarrhea cases and healthy controls. The molecular analysis of SaV and NoV (GI, GII) showed that NoV GII was the major subtype of HuCV, and GII.4 was the most predominant genogroup detected in diarrhea patients. In addition, GII.17 variant emerged in diarrhea cases

Eleven strains of HuCV were found in healthy controls. One (9.1 %) case was classified as NoV GI, which was a GI.3 genotype, and 10 (90.9 %) cases were classified as NoV GII, which were divided into GII.4 (*n* = 6, 60.0 %), GII.12 (*n* = 3, 30.0 %), and GII.5 (*n* = 1, 10.0 %). Of the six strains of GII.4, four (80.0 %) strains were of the GII.4 Sydney 2012 variant and two (20.0 %) were of the GII.4-2006b variant. The GII.4 Orleans 2009 variant was not found in healthy controls (see Fig. 1).

## Discussion

Acute diarrhea is still a serious public health problem, and is the second leading cause of morbidity and mortality among children below five years of age worldwide [1, 2]. NoV is one of the major enteropathogen causes of sporadic diarrhea in all age groups [10]. In China, most studies of acute gastroenteritis including HuCV infection have focused on children below five years of age [22, 26]. Few studies have been conducted to clarify the HuCV epidemiological characteristics in children and adults [27, 28], especially in developing regions [26]. This study provided new data on acute diarrhea cases infected with HuCV in Yunnan Province, China. The present study described the detection rate, epidemiological characteristics of HuCV subtypes, and genetic diversity of NoV genotypes.

In this study, NoV GII was the most common genogroup detected among diarrhea cases and healthy controls, and GII.4 was the most prevalent genotype. The GII.4 Sydney 2012 variant was still the most prevalent one both in patients and controls of all ages. Although the GII.17 genotype emerged at the end of 2014 and was most prevalent in outbreaks of diarrhea in some Asian countries and the United States [29–33], the GII.17 variant did not replace the GII.4 Sydney 2012 variant in sporadic diarrhea cases in the study. This finding was in accordance with other studies conducted in China [28, 34], which suggested that the GII.4 Orleans 2009 variant did not replace the GII.4-2006b variant and was not prevalent in China [28, 34]. The molecular epidemiology of NoV in China was that the GII.4 Sydney 2012 variant directly replaced the GII.4-2006b variant and has become the predominant variant since 2012 [34–36]. Moreover, this epidemic’s characteristics were not accordance with findings from other studies conducted in some European and American countries, where the GII.4 Orleans 2009 variant has successfully replaced the GII.4-2006b variant since 2009, and the GII.4 Sydney 2012 variant has replaced the GII.4 Orleans 2009 variant since 2012 [6, 34].

In this study, NoV GII was one of the diarrhea-associated pathogens in all age groups, but NoV GII did not cause acute diarrhea in individuals over 65 years. Some studies have also suggested that NoV disease risk among older adults has decreased in upper-middle and high-income countries [37]. Other studies showed that NoV GII was a diarrhea-associated pathogen and the attributable risk of NoV GII was located the third rank among all enteric pathogens, which was only lower than that of rotavirus and *Shigella* spp. [38]. However, other studies showed that NoV GII was not an enteropathogen associated with diarrhea [39]. This conclusion might be attributable to selection bias in the samples sources, which were all from pediatric emergency departments.

NoV GII infection had relative low risk in children under five years (seen in Table 1). Several factors may explain this phenomenon: on the one hand, immune defenses (such as milk IgA antibodies) might protect against NoV GII infection in children below six months of age [40, 41]; on the other hand, non-specific properties (such lactoferrin and enterotoxin-binding oligosaccharides present in artificial milk powder, which is the most common food for children) might prevent the adherence of NoV GII to mucosal invasion to reduce the virus count or toxicity [40, 42]. These factors might also interpreted that the healthy carrier rate of NoV GII in non-diarrhea subjects below five years of age (5.5 %) was higher than that of healthy controls above five years of age (1.5 %). Mathematical modeling also proved that protection against acute diarrhea could be acquired in early life and led more frequently to NoV asymptomatic infection [43].

The study showed that NoV GII was the prominent HuCV subtype, but NoV GI and SaV were rarely found in diarrhea patients. The composition of these three viruses was similar to results from other studies, which showed that NoV GII accounted for more than 90 % of all HuCV-positive cases in China [34]. In this study, GII.4 was preponderant genotype in all genotypes of NoV GII, which was in accordance with the epidemic characteristics of GII.4 in other studies [44], but the constituent ratio of GII.4 in all NoV GII genotypes was less than that found in other studies conducted in the period of 2008–2009 in China [28]. The reason for this might be that GII.4 was prevalent for several years, while the GII.17 has been emergent and prevalent only since 2014 [29].

The prevalence of NoV was 10.7 % in acute gastroenteritis in patients of all ages in this study, which was lower than the global detection rate (18.0 %), but was close to the prevalence in high-mortality countries (14.0 %) [45]. The low prevalence of NoV might suggest that other enteropathogens such as bacterial and parasitic pathogens played a prominent role in diarrhea disease [10]. Acute gastroenteritis cases due to NoV had mild diarrhea symptoms, thus many NoV-related diarrhea cases might not seek medical treatment. This might explain why the prevalence of NoV in community cases (24.0 %) was higher than that found in outpatient cases (17.0 %) [10]. Diarrhea cases in our study were all selected from outpatient departments, which might be the reason for the low prevalence of NoV in this study. The prevalence of NoV in this study was lower than that found in Shanghai, China [27]. This difference might be caused by the different food habits among residents in different areas; shellfish, which is easily infected with NoV [46], was one of the common foods for residents in Shanghai, which is near the Yangtze river and the sea, but shellfish is not commonly eaten in inland regions. Food species might be one of the important reasons for the variation of NoV in different regions.

There were no significant differences in NoV GII prevalence across several age groups, which might suggest that age had no significant influence on NoV infection. Other studies led to similar conclusions [28, 34]. The reasons might be that NoV had great diversity and a lack of long-term immunity, hence human immunity to NoV was not stable and repeated infection might occur throughout life [8]. But the prevalence of GII was slightly higher in children aged below five years with diarrhea and in elderly patients. This finding was in accordance with some other reports [43, 47]. NoV tended to infect these subgroups because they have relatively low immunity to NoV [20]. One interesting finding of this study was that diarrhea cases in children below two years of age and elderly patients were susceptible to the GII.17 genotype, which might be explained by the weak immune system of these two age groups and the fact that the newly emergent GII.17 had no immune barrier among the population.

In this study, the GII.17 variant emerged in diarrhea patients. This finding was in accordance with other studies. The GII.17 variant was the prominent genotype in sporadic diarrhea patients, the detection rate of which in diarrhea cases was up to 15.0 %, which was higher than the prevalence of the GII.4 Sydney 2012 strain in sporadic diarrhea patients in Jiangsu Province, China, from the end of 2014 onwards [29]. The novel pathogen GII.17 variant was found in diarrhea patients in the study, which benefitted from the web-based information system for disease control and prevention in China [48]. In addition, laboratory-based pathogen diagnosis was conducted to find novel pathogens or variant strains [49, 50]. In the meantime, a syndrome-based surveillance system has also been important in supporting the detection of variations in the intestinal pathogen spectrum and the emergence of the new pathogen or variant strain in China [50, 51].

### Limitations of this study

There were several limitations on our research. Firstly, the subjects were mainly selected from urban areas, and in addition, the diarrhea cases were all selected from outpatient department, which might lead to selection bias in respect of the epidemiological characteristics of HuCV. Secondly, the number of healthy controls was small, hence the OR of the HuCV and NoV genogroups were not highly reliable. Thirdly, genetic characterization of NoV was only based on partial polymerase regions in this study. However, the genotyping of NoV based on only capsid regions or on both regions might provide different results. Therefore, further study involving diarrhea cases selected from urban, rural, outpatient, and inpatients departments will should be conducted to ensure accurate assessment of the NoV disease burden. A 1:1 match case-control study will be a good choice. Meanwhile, genotyping of NoV based on both capsid regions and RNA-dependent RNA polymerase regions should be conducted in order to enhance the accuracy and effectiveness of NoV GII genotyping.

## Conclusions

In summary, the results show that NoV is one of the major causes of diarrhea, that GII.4 is still the predominant strain, and that GII.17 has emerged in sporadic diarrhea cases and might become a predominant strain, which will lead to more cases and outbreaks of diarrhea in the near future. This will provide new obstacles to preventing NoV-associated diarrhea. Therefore, monitoring the prevalence trends and evolution of GII.17 is necessary.

## Abbreviations

CI: Confidence interval
GI: Genogroup I
GII: Genogroup II
HuCV: Human caliciviruses
NoV: Norovirus
OR: Odds ratio
RT-PCR: Reverse transcriptase polymerase chain reaction
SaV: Sapovirus
